# Sortase-mediated assembly and surface topology of adhesive pneumococcal pili

**DOI:** 10.1111/j.1365-2958.2008.06396.x

**Published:** 2008-09-17

**Authors:** Stefan Fälker, Aaron L Nelson, Eva Morfeldt, Kristina Jonas, Kjell Hultenby, Johannes Ries, Öjar Melefors, Staffan Normark, Birgitta Henriques-Normark

**Affiliations:** 1 Department of Bacteriology, Swedish Institute for Infectious Disease ControlSE-171 82 Solna, Sweden; 2 Department of Microbiology, Tumour Biology and Cellbiology, Karolinska InstitutetSE-171 77, Stockholm, Sweden; 3 Clinical Research Center, Department of Laboratory Medicine, Karolinska InstitutetSE-141 86, Huddinge, Sweden

## Abstract

The *rlrA* genetic islet encodes an extracellular pilus in the Gram-positive pathogen *Streptococcus pneumoniae*. Of the three genes for structural subunits, *rrgB* encodes the major pilin, while *rrgA* and *rrgC* encode ancillary pilin subunits decorating the pilus shaft and tip. Deletion of all three pilus-associated sortase genes, *srtB*, *srtC* and *srtD*, completely prevents pilus biogenesis. Expression of *srtB* alone is sufficient to covalently associate RrgB subunits to one another as well as linking the RrgA adhesin and the RrgC subunit into the polymer. The active-site cysteine residue of SrtB (Cys 177) is crucial for incorporating RrgC, even when the two other sortase genes are expressed. SrtC is redundant to SrtB in permitting RrgB polymerization, and in linking RrgA to the RrgB filament, but SrtC is insufficient to incorporate RrgC. In contrast, expression of *srtD* alone fails to mediate RrgB polymerization, and a *srtD* mutant assembles heterotrimeric pilus indistinguishable from wild type. Topological studies demonstrate that pilus antigens are localized to symmetric foci at the cell surface in the presence of all three sortases. This symmetric focal presentation is abrogated in the absence of either *srtB* or *srtD*, while deletion of *srtC* had no effect. In addition, strains expressing *srtB* alone or *srtC* alone also displayed disrupted antigen localization, despite polymerizing subunits. Our data suggest that both SrtB and SrtC act as pilus subunit polymerases, with SrtB processing all three pilus subunit proteins, while SrtC only RrgB and RrgA. In contrast, SrtD does not act as a pilus subunit polymerase, but instead is required for wild-type focal presentation of the pilus at the cell surface.

## Introduction

Pili, or fimbriae, are a diverse set of fibrous extracellular appendages expressed by bacteria to facilitate interactions with host cells and other bacteria (Hultgren *et al*., 1996; Telford *et al*., 2006). Pili expressed by Gram-positive bacteria were considered an unusual feature found only in the dental pathogens *Actinomyces* spp. (Yeung and Ragsdale, 1997; Yeung *et al*., 1998; Mishra *et al*., 2007) until 2003, when Ton-That *et al*. described pilus expression in *Corynebacterium diphtheriae* (Ton-That and Schneewind, 2003). Pili have since been described in many Gram-positive bacteria, including group A streptococci (Mora *et al*., 2005), group B streptococci (Lauer *et al*., 2005), *Streptococcus pneumoniae* (Barocchi *et al*., 2006), *Enterococcus faecalis* (Nallapareddy *et al*., 2006) and *Bacillus cereus* (Budzik *et al*., 2007), and probable pilus loci were identified by genome sequencing of additional *Streptococcus* spp. (Osaki *et al*., 2002; Xu *et al*., 2007). Through this body of work, a picture of the genetic and biochemical characteristics of pili in Gram-positive bacteria has emerged.

Pili of Gram-positive bacteria are antigenic in humans *in vivo* (Mora *et al*., 2005) and protective of immunized animals in the laboratory (Rosini *et al*., 2006; Gianfaldoni *et al*., 2007). Pilus expression increases pathogenicity in animal models (Hava and Camilli, 2002; Abbot *et al*., 2007), and enhances adhesion to epithelial cells (Barocchi *et al*., 2006; Dramsi *et al*., 2006; Maisey *et al*., 2007). The pili are composed of covalently polymerized subunits, effectively creating one molecule, with a mass often exceeding 10^6^ Da on Western blots (WB) (Ton-That and Schneewind, 2003). Hence, once assembled, pili have been suggested to be static structures, incapable of disassembly or modification (Ton-That and Schneewind, 2004; Scott and Zahner, 2006; Telford *et al*., 2006).

All pilin proteins in Gram-positive organisms possess a so-called LPXTG-type sorting signal (Schneewind *et al*., 1992), which has been shown to be present in proteins anchored to the peptidoglycan matrix of the cell wall by the ‘house-keeping’ enzyme Sortase A (SrtA) in staphylococci (Marraffini *et al*., 2006). Sortase A cleaves the LPXTG motif between the threonine (T) and glycine (G) and subsequently the C-terminal threonine is linked to a cysteine residue of the sortase (Marraffini *et al*., 2006; Scott and Barnett, 2006). Eventually the protein is incorporated into the cell wall by formation of an amide bond between the C-terminal threonine of the surface protein and the stem peptide of the peptidoglycan. Recently, it was shown that the activity of house-keeping sortase enzymes from staphylococci and bacilli could be inhibited by a covalent modification of their active-site cysteine (Maresso *et al*., 2007).

Pilin genes in Gram-positives are found in islets with genes encoding pilus-associated sortases (Ton-That and Schneewind, 2003; 2004; Scott and Zahner, 2006; Telford *et al*., 2006), enzymes homologous to SrtA recognizing distinct sequence signatures (Comfort and Clubb, 2004; Dramsi *et al*., 2005). These enzymes are essential in the assembly of mature pili in all cases studied thus far (Ton-That and Schneewind, 2003; Mora *et al*., 2005; Dramsi *et al*., 2006; Gaspar and Ton-That, 2006; Nallapareddy *et al*., 2006; Rosini *et al*., 2006; Swierczynski and Ton-That, 2006; Budzik *et al*., 2007), and likely act as pilus subunit polymerases (Scott and Zahner, 2006). The sorting signal has been shown to be necessary for incorporation of pilin subunits into polymeric pilus fibres (Ton-That and Schneewind, 2003), by catalysing the formation of a peptide bond between the threonine in the cell wall signalling motif of pilins and a lysine residue of another pilin subunit.

Our laboratory described the pilus of the Gram-positive human respiratory commensal and pathogen, *S. pneumoniae* (or pneumococcus) (Barocchi *et al*., 2006), and has recently explored the importance of the pilus in natural pneumococcal populations (Sjostrom *et al*., 2007). The pneumococcal *rlrA* pilus islet possesses seven genes, several of which have been shown to be necessary in animal models of colonization and disease (Hava and Camilli, 2002). Three of these genes, *rrgA*, *rrgB* and *rrgC*, encode structural subunits, and RrgB has been predicted to be the major pilin by both similarity to known pilins in other Gram-positive bacteria (Ton-That and Schneewind, 2003), genetic analysis (LeMieux *et al*., 2006), and immunoelectron microscopy (iEM) studies (Hilleringmann *et al*., 2008), and data presented in this work confirm this assertion. Assembly of the pneumococcal pilus would appear to be a more complex process than seen in homologous pili, as all other known pilus islands contain one or two sortase genes, while *S. pneumoniae* possesses three, *srtB*, *srtC* and *srtD*.

In this study we sought to clarify the relative contributions of the three genes encoding pilus-associated sortases in the *S. pneumoniae* pilus islet. We show that SrtB is important in RrgB polymerization and the only sortase that may incorporate the minor pilin subunit RrgC into the polymer, a process dependant on the active-site cysteine in SrtB. Furthermore, it has been suggested that discrete sites of proteins secretion (Rosch and Caparon, 2004) and surface sorting (DeDent *et al*., 2007) exist in Gram-positive organisms, and that these systems might be topologically co-ordinated (Hu *et al*., 2007). We therefore hypothesized that pneumococcal pili might be distributed non-homogenously on the surface of wild-type organisms. Indeed, discrete non-homogenous topological distribution of pili antigen was observed and described in this report. We further demonstrate that SrtD plays a central role in this ordered localization, despite lacking capacity to catalyse pilin polymerization.

## Results

### Ultrastructural analyses of the pneumococcal pilus

**Fig. 1:**
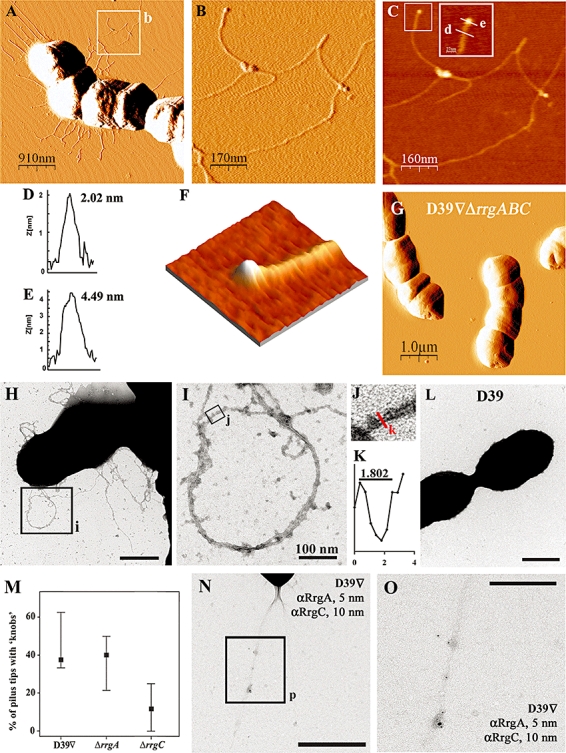
Structural characterization of the pneumococcal pilus by AFM and EM. Pili on D39∇(*rlrA-srtD*) cells were visualized by AFM (A–G) or by transmission EM (H–L). A–E. Low-magnification (A, 910 nm scale bar shown) and high-magnification (B, 170 nm scale bar) AFM images of pili, with a matching topographic projection shown in (C) (with 160 nm scale bar). Insert shows a terminal pilus, at which two height measurements were taken over the length of lines shown, with height (*z*-axis) deflection plots in nm in (D) and (E). Note that the pilus shaft diameter is estimated to be approximately 2 nm, assuming radial symmetry. Also note that the terminal ‘tip’ is estimated to be approximately 4.5 nm, suggesting different or additional structures than observed in the pilus shaft. F. Thickness projection and magnification of a subset of (C), showing the thickness projection of the structure measured in (D) and (E). G. D39∇(*rlrA-srtD*)Δ*rrgABC* serves as a negative-control strain and does not generate pili (1.0 μm scale bar). H and I. Low-magnification (H, scale bar 500 nm) and high-magnification (I, scale bar 100 nm) images of pili by EM. J. A digital magnification of a subset of (I) showing a pilus fibre with a red line indicating a measurement site. K. Greyscale value (arbitrary units) is plotted against distance in nm over the line shown in (J), indicating that this pilus fibre is approximately 1.8 nm in diameter. L. D39, the parental strain that lacks the pilus islet transgene inserted into D39∇(*rlrA-srtD*), served as a negative control, and did not produce detectable extracellular fibres (scale bar 500 nm). M. We tested whether RrgC composed the ‘tip knobs’ by determining tip knobs per pilus fibre ratios in high-magnification AFM fields, comparing strains D39∇(*rlrA-srtD*) (‘D39∇’), D39∇(*rlrA-srtD*)Δ*rrgA* (‘Δ*rrgA*’) and D39∇(*rlrA-srtD*)Δ*rrgC* (‘Δ*rrgC*’). The *rrgC* mutant exhibited fewer ‘tip knobs’ per fibre, supporting a model whereby RrgC is the predominant species in the pilus tip complex. N and O. Double labelling for both antigens permitted identification of ‘patches’ including both RrgA and RrgC in D39∇ at both low- (N, 500 nm scale bar) and high-magnification (O, 200 nm scale bar). P = part of figure N is shown in higher magnification in O.

Pili expressed by D39∇(*rlrA-srtD*) (called ‘D39∇’ hereafter), a serotype 2 strain that carries the complete pilus islet of the sequenced strain TIGR4 (‘T4’) (Barocchi *et al*., 2006), were found to be at least 0.5 μm in length, often 1.5 μm or greater, whether examined by atomic force microscopy (AFM) (Fig. 1A–G) or examined by transmission electron microscopy (EM) (Fig. 1H–L). Fibres were frequently tangled, or web-like, with two or three fibres wrapping around each other (Fig. 1A–C, H and I). High-magnification AFM examination revealed thin fibres with bulbous decorations both internal to the fibre (‘internal knobs’) and at the tip (‘tip knobs’) (Fig. 1B–C and F). AFM permits accurate measurements of subject thickness, and such a study yielded a pilus fibre thickness of 2.09 ± 0.36 nm (*n* = 23 independent determinations) (examples in Fig. 1C–E). The terminal decorating structures are notably thicker than pilus fibres (Fig. 1C–F). A three-dimensional projection of a pilus fibre and tip structure is shown in Fig. 1F and illustrates how the tip is raised, suggesting the existence of a protein complex (Fig. 1F). Negatively stained pili were examined by high-magnification EM (Fig. 1I) and digital enhancement (Fig. 1J), revealing stain deposition along the edges of thin, stain-impermeable fibres. These fibres were estimated to be 2.14 ± 0.38 nm wide (*n* = 36 independent determinations) (example in Fig. 1J and K). Ultrastructural studies were limited to D39∇, as a larger fraction of those cells were piliated compared with T4 (Fig. S1).

### RrgB is the major pilin, and RrgA and RrgC form decorating structures

To understand the genetic determinants of the pilus structure, we generated strains with inactivating insertion-deletions in each of the three structural genes of the *rlrA* pilus islet, *rrgA*, *rrgB* and *rrgC*. WB analysis of corresponding mutants in strains T4, D39∇ and BHN100 yielded qualitatively identical results, and results from T4 and D39∇ are presented in *Supporting information* (Fig. S2).

Immunological and genetic studies suggest that RrgB is the major pilin composing the pilus shaft (Barocchi *et al*., 2006; LeMieux *et al*., 2006). Deletion of *rrgB* abolished expression of high-molecular-weight (> 250 kDa) immunoreactive ladders in the cell wall-associated protein fraction of T4 and D39∇ (Fig. S2), and *rrgB*-deficient D39∇ organisms lacked visible pili when examined by EM and AFM (Fig. S3). Mutants in *rrgA* or *rrgC* in T4 produced high-molecular-weight RrgB-positive ladders (Fig. S2) with pili confirmed by EM and AFM (Fig. S3). Inactivation of *rrgC* did not inhibit incorporation of RrgA into pili by WB of T4 and D39∇ strains (Fig. S2) and iEM of D39∇Δ*rrgC* (Fig. S3), and inactivation of *rrgA* did not prevent incorporation of RrgC (Figs S2 and S3). Thus, *rrgB*, but not *rrgA* or *rrgC*, is required for formation of pili, and RrgB is the ‘major’ pilin polymerized to form the pilus shaft, which can be decorated with ancillary subunits independently of each other.

We frequently observed RrgC at the tips of antibody-labelled pili by EM studies (Barocchi *et al*., 2006; LeMieux *et al*., 2006), suggesting that RrgC might compose the ‘tip knobs’ observed by AFM analysis (Fig. 1F). To test this, we compared fractions of pili with ‘tip knobs’ in piliated D39∇, D39∇Δ*rrgA* and D39∇Δ*rrgC.* D39∇ (37.5% median, 33.3% minimum, 62.5% maximum) and D39∇Δ*rrgA* (40.0% median, 21.4% minimum, 50% maximum) preparations exhibited tip ‘knob’ frequencies that were statistically indistinguishable from one another, while the tip ‘knob’ frequency of D39∇Δ*rrgC* was significantly reduced (11.5% median, 0.0% minimum, 25.0% maximum) (Fig. 1M), suggesting an important role for RrgC in forming pilus tip ‘knobs’.

Furthermore, we found RrgC and RrgA in clusters along the length of the pilus fibre. By double-labelling studies we could show that both RrgA and RrgC are found at identical clusters along the pilus shaft (Fig. 1N and O), although our analysis does not permit determination of the fraction of RrgA or RrgC found together or separate. We hypothesized that these RrgA-positive, RrgC-positive clusters might be the ‘internal knobs’ observed by AFM (Fig. 1B and C). Genetic inactivation of either *rrgA* or *rrgC* independently reduced the number of ‘internal knobs’, but not to a statistically significant degree (data not shown). Thus, it is likely that ‘internal knobs’ along the RrgB pilus stalk can be made of RrgA and RrgC separately, or RrgA and RrgC together. Further, the pilin-specific sortases must be capable of linking RrgA, RrgB and RrgC to RrgB, as well as RrgC to RrgA. A direct interaction between RrgA and RrgC is supported by detection of a 130 kDa band in WB with RrgA and RrgC, but not with RrgB-specific antibodies (Fig. S1, most prominently seen in Δ*rrgB* strains). A mass of 130 kDa is the predicted combined mass of RrgA and RrgC.

### The contribution of pilus-associated sortases to pilus biogenesis

**Fig. 2:**
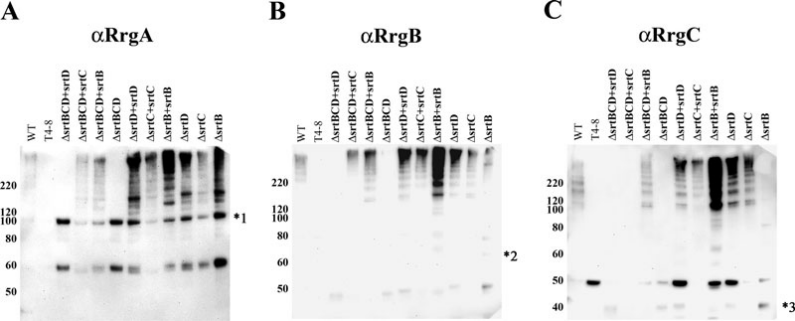
Redundant role of individual sortases in pilus biogenesis. Production of polymeric high-molecular-weight cell wall-associated pili in isogenic T4 mutants was evaluated by immunoblotting for RrgA (A), RrgB (B) and RrgC (C). In all cases, cell wall proteins were separated by gradient SDS-PAGE, transferred to PVDF and probed. Approximate molecular weights in kDa are indicated on left, based on marker proteins. A. Immunoblotting for RrgA in wild-type T4 (‘WT’), T4Δ*rlrA-srtD* (‘T4-8’), the *trans*-complemented *srtBCD* triple-mutant strains T4Δ*srtBCD*+*lacE*::*srtD* (‘Δ*srtBCD*+*srtD*’), T4Δ*srtBCD*+*lacE*::*srtC* (‘Δ*srtBCD*+*srtC*’) and T4Δ*srtBCD*+*lacE*::*srtB* (‘Δ*srtBCD*+*srtB*’), the triple-mutant T4Δ*srtBCD* (‘Δ*srtBCD*’), the *trans*-complemented single sortase mutant strains T4Δ*srtD*+*lacE*::*srtD* (‘Δ*srtD*+*srtD*’), T4Δ*srtC*+*lacE*::*srtC* (‘Δ*srtC*+*srtC*’) and T4Δ*srtB*+*lacE*::*srtB* (‘Δ*srtB*+*srtB*’), T4Δ*srtD* (‘Δ*srtD*’), T4Δ*srtC* (‘Δ*srtC*’) and T4Δ*srtB* (‘Δ*srtB*’). The predicted 90 kDa RrgA monomer is indicated by asterisk 1. B and C. (B) Immunoblotting for RrgB (67 kDa RrgB monomer is indicated by asterisk 2), and immunoblotting for RrgC (predicted 38 kDa RrgC monomer is indicated by asterisk 3) (C), with samples loaded as in (A). Lack of polymer formation in T4Δ*srtBCD* shows that at least one of the three pilus-associated sortases, SrtB, SrtC or SrtD, is required for pilus biogenesis. SrtB and SrtC are sufficient for pilin polymerization, as shown by T4Δ*srtBCD*+*lacE*::*srtB* and T4Δ*srtBCD*+*lacE*::*srtC*, while SrtD is not capable of pilin polymerization (T4Δ*srtBCD*+*lacE*::*srtD*). Finally, these data demonstrate that SrtB is necessary to conjugate RrgC to a pilus polymer.

A mutant in all three sortases, T4Δ*srtBCD*, was completely deficient in production of pili by WB (Fig. 2A–C, lanes 6). Thus, like pilus loci of other Gram-positive organisms, the islet-associated sortases are essential in the assembly of mature pneumococcal pili. T4Δ*srtBCD* did express 90 kDa predicted RrgA monomers (Fig. 2A), which were present in the cell wall even in the absence of pilus-associated sortase expression.

Individual insertion-deletion mutants in each of the three sortases were then generated. Mutation of either *srtC* or *srtD* individually did not abrogate the incorporation of RrgA, RrgB or RrgC (Fig. 2A–C, lanes 10 and 11) into the pilus. However, deletion of *srtB* completely abrogated the incorporation of RrgC into the pilus polymer (Fig. 2C, lane 12), although this strain generated abundant RrgA- and RrgB-positive pili (Fig. 2A and B). Furthermore, abundant RrgC was detectable in whole cultures, cellular and supernatant fractions of T4Δ*srtB* (data not shown), demonstrating that the *rrgC* gene was expressed. These data were confirmed using a *trans-*complemented mutant strain, T4Δ*srtB*+*lacE*::*srtB*, which was shown to produce RrgC-positive pili qualitatively indistinguishable from wild type (Fig. 2C, lane 9). Hence, sortase B is necessary for the covalent incorporation of RrgC into pili polymers.

To determine if sortase B alone can polymerize RrgB, we transformed the triple sortase mutant T4Δ*srtBCD* with a construct providing a second copy of *srtB* intact *in trans*. The resulting strain, T4Δ*srtBCD*+*lacE*::*srtB*, was sufficient to assemble heteropolymeric RrgA-, RrgB- and RrgC-positive pili (Fig. 2A–C, lanes 5).

To analyse the specific roles of SrtC and SrtD for pilus assembly, strains expressing only SrtC (T4Δ*srtBCD*+*lacE*::*srtC*) or *srtD* (T4Δ*srtBCD*+*lacE*::*srtD*) were generated by similar means. T4Δ*srtBCD*+*lacE*::*srtC* assembled RrgA- and RrgB-heteropolymers with no detectable RrgC (Fig. 2A–C, lanes 4). Thus, the phenotype of T4Δ*srtBCD*+*lacE*::*srtC* is qualitatively identical to that of T4Δ*srtB*. To determine if SrtD plays an independent role in the assembly of pneumococcal pili, WB of cell wall-associated material from T4Δ*srtBCD*+*lacE*::*srtD* were analysed. T4Δ*srtBCD*+*lacE*::*srtD* expressed no detectable dimers or polymers of RrgA, RrgB or RrgC (Fig. 2A–C, lanes 3). Thus, SrtD possesses no independent polymerization function under the conditions tested.

### Sortase expression contributes to pilus surface topology

**Fig. 3:**
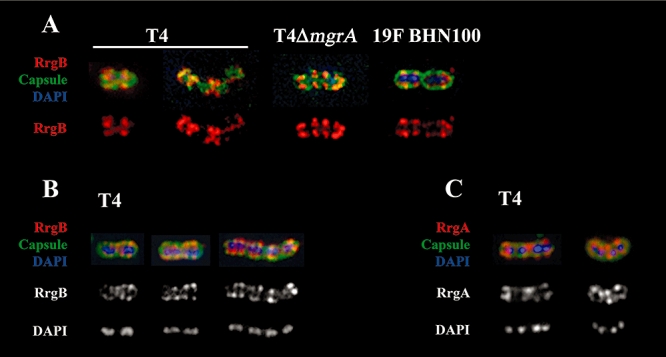
Topology of pilus antigen in *S. pneumoniae*. Immunofluorescence (IF) microscopy of piliated pneumococci from two clonal lineages reveals non-homogenous pilus antigen topology, consistent with ‘bands’ or ‘rings’. EM and AFM preparations were adapted for IF staining for anti-RrgB (A and B) and anti-RrgA (C) antibodies, shown in red in these images. Cells were also stained with antibodies against the polysaccharide capsule, in green, and nucleoids were stained with DAPI, in blue. Examples of RrgB topology in the T4 and serotype 19F strain BHN100 clonal lineages were visualized by confocal microscopy (A). T4Δ*mgrA* is a pilus-overexpressing strain described elsewhere (Hemsley *et al*., 2003). Multiple examples of RrgB (B) and RrgA (C) topology imaged by conventional microscopy are shown for T4. RrgB is found in discretely concentrated foci, often paired across the division plane, or in a ring encircling the cell. Similar examples of RrgB and RrgA topology in 19F BHN100 are shown in Fig. S5.

Wild-type pneumococci were stained by immunofluorescence (IF) for the localization of pilus antigens on the bacterial cell surface. RrgB was found in discrete, symmetrically arranged foci on the cell surface of wild-type T4 and 19F BHN100 cells, and on T4 organisms with a mutation in the negative regulator, *mgrA* (Hemsley *et al*., 2003; Barocchi *et al*., 2006), by confocal (Fig. 3A, red) and conventional microscopy (Fig. 3B). This localized pattern of the RrgB antigen is in contrast to other surface antigens, such as the polysaccharide capsule, which exhibited uniform distribution (Fig. 3A, green). Nor is this effect an artefact of the antibody employed for staining, as no staining was observed in a strain lacking pili nor when the primary antibody was excluded (Fig. S4), and further, a similar staining pattern was observed for RrgA staining (Fig. 3C and Fig. S5). It appeared as if each DAPI-positive nucleoid is accompanied by a pair of foci on either side of the cell, or, possibly, a ring across the plane of cellular fission. Despite attempts with multiple antibodies, we were unable to stain pneumococci for RrgC, suggesting that there is insufficient RrgC antigen per cell to detect by IF (data not shown). RrgB and RrgA distribution in BHN100 revealed topology identical to T4 and was, perhaps, even more discrete (Fig. S6A).

**Fig. 4:**
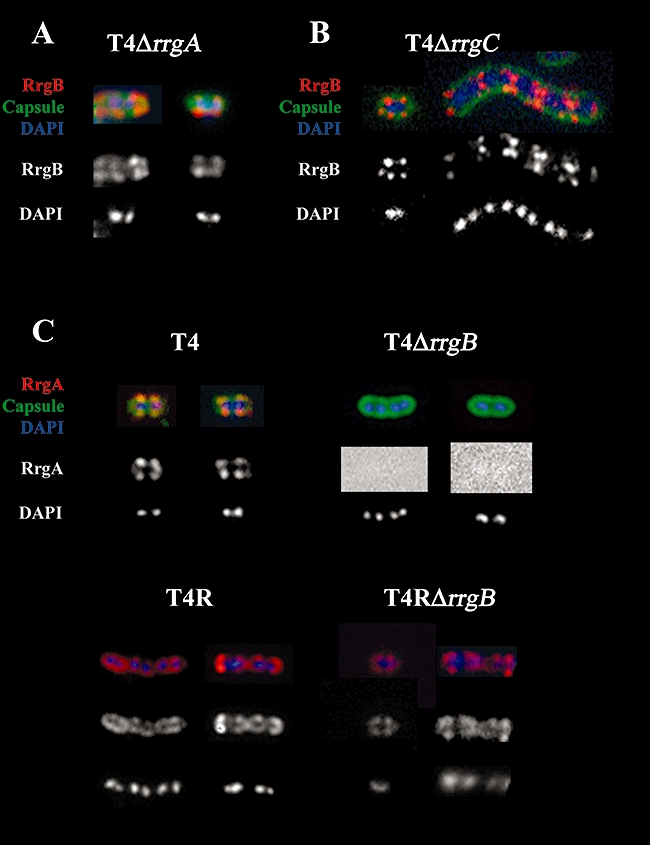
Pilus topology is not determined by pilins. RrgB topology was studied in T4Δ*rrgA* (A) and T4Δ*rrgC* (B), and representative images show RrgB immunostaining in red, capsule immunostaining in green and the nucleoid stained with DAPI in blue. Note that in both mutants, RrgB is found in symmetric foci, similar to observations in wild-type T4 (Fig. 3). RrgA is not detectable in T4Δ*rrgB* (C, top right), making it impossible to determine if RrgA topology is determined by RrgB expression. We hypothesized that RrgA is obscured by the capsule, and therefore evaluated RrgA topology in an unencapsulated mutant of T4, T4R (C, bottom left), and an *rrgB* mutant of T4R, T4RΔ*rrgB* (C, bottom right). RrgA is preserved as symmetric rings in T4R, although the topology is less discrete than observed in the presence of a capsule (C, top versus bottom left), possibly due to RrgA monomers in the wall. We therefore conclude that RrgA topology is not dependent on *rrgB* expression. Furthermore, this demonstrates that the pilus shaft functions to extend RrgA and RrgC monomers beyond the capsule. Therefore, pilin antigens detected by IF in an encapsulated organism are not monomers, and are instead higher-order multimeric species.

We hypothesized that the localized surface distribution of pilin proteins could be due to biochemical information within the subunits themselves necessary to self-organize. However, RrgB organization is preserved in T4Δ*rrgA* (Fig. 4A) and T4Δ*rrgC* (Fig. 4B), and RrgA topology in T4Δ*rrgC* is comparable to wild type (Fig. S5). However, RrgA was not detectable by IF in T4Δ*rrgB* (Fig. 4C), despite abundant production of RrgA in putative monomers and RrgA–RrgC heterodimers (Fig. S1), suggesting that the capsule obscures low-molecular-weight forms of RrgA. To test this hypothesis, *rrgB* was inactivated in an unencapsulated mutant of T4, T4R (Fernebro *et al*., 2004), to generate strain T4RΔ*rrgB*. RrgA topology in T4RΔ*rrgB* was identical to that of the parental unencapsulated, piliated T4R, with notable bands of antigen across the cellular surface (Fig. 4C). These data emphasize that pilin genes themselves do not determine topological distribution of pilins.

**Fig. 5:**
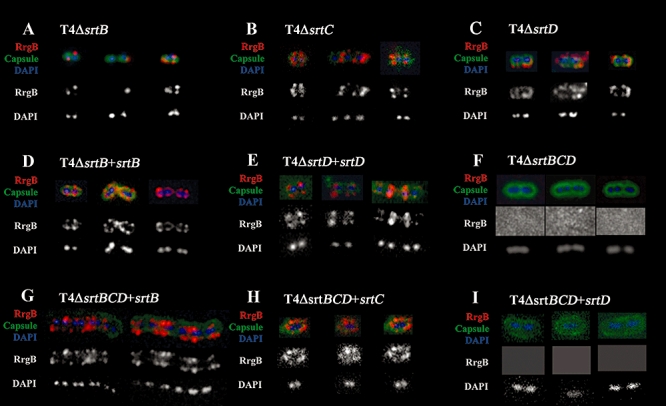
Roles of pilus-associated sortases in pilus topology. RrgB topology was examined in isogenic sortase mutants of T4. RrgB immunostaining is shown in red, capsule immunostaining in green and the nucleoid stained with DAPI in blue. A. RrgB focus formation was observed in T4Δ*srtB*, but the foci are not co-ordinated into pairs or rings. B. T4Δ*srtC* exhibited RrgB topology indistinguishable from wild-type cells. C. T4Δ*srtD* exhibited a large number of small, poorly organized RrgB foci diffusely distributed along cell chains. D. Complementation of *srtB in trans*, strain T4Δ*srtB*+*lacE*::*srtB* (‘T4Δ*srtB*+*srtB*’), restores discrete symmetrical RrgB distribution, proving the necessity of SrtB in determining pilus topology. E. The necessity of SrtD in determining pilus topology was proven by complementation of *srtD in trans*, strain T4Δ*srtD*+*lacE*::*srtD* (‘T4Δ*srtD*+*srtD*’). F. Inactivation of all three pilus-associated sortases in T4Δ*srtBCD* results in failure to detect any RrgB on the surface of the pneumococci. G. Insertion of a second copy of *srtB* on a transgene in strain T4Δ*srtBCD*+*lacE*::*srtB* (‘T4Δ*srtBCD*+*srtB*’) restores detectable RrgB on the surface of T4. Moreover, this strain displays diffuse RrgB foci, like T4Δ*srtD*, supporting a role for SrtD in organizing pilus antigen. H. Strain T4Δ*srtBCD*+*lacE*::*srtC* (‘T4Δ*srtBCD*+*srtC*’) expressing only *srtC* shows a similar phenotype like T4Δ*srtD* with diffuse RrgB foci. I. No RrgB can be detected after expression of *srtD in trans* in a triple-sortase mutant (‘T4Δ*srtBCD*+*srtD*’). The effect of *srtB* and *srtD* mutations on RrgA topology in T4 is similar, and shown in Fig. S5. Moreover, *srtB* and *srtD* mutations have similar effects on RrgB and RrgA topology in 19F BHN100 (Fig. S6).

To investigate putative roles of the sortases for pilus topology, we determined the distribution of pilus subunits in sortase-deficient strains. Surprisingly, the regular distribution of RrgB (Fig. 5C) and RrgA (Fig. S5) was severely impaired in T4Δ*srtD*, which displayed numerous, small foci without any apparent organization. Disruption of *srtB* also affected pilus topology, as T4Δ*srtB* displayed smaller number of RrgB (Fig. 5A) and RrgA (Fig. S5) foci with inconsistent or irregular cellular location(s). Complementation of the disrupted *srtB* and *srtD* restored normal distribution of RrgB (Fig. 5D and E) and RrgA (Fig. S5), confirming the specific roles of *srtB* and *srtD* for proper surface location. In contrast, T4Δ*srtC* exhibited RrgB topology identical to wild-type T4 (Fig. 5B). Notably, RrgB (Fig. S6B) and RrgA (Fig. S6C) topology is also disturbed in BHN100Δ*srtB* and BHN100Δ*srtD* when compared with the wild type. Thus, disruption of *srtB* or *srtD* perturbs normal surface distribution of pilins in two unrelated clonal lineages.

Inactivation of all three pilus-associated sortases in strain T4Δ*srtBCD* prevented the detection of surface RrgB (Fig. 5F), in agreement with WB analysis (Fig. 2). Although WB studies indicate that monomeric RrgA is found in the cell wall fraction of T4Δ*srtBCD* (Fig. 2A), neither subunit protein was detectable by IF (Fig. 5F, and data not shown). We hypothesized that the polysaccharide capsule of the pneumococcus, which prevents antibody-mediated detection of surface proteins, obscures monomeric RrgA, as suggested by studies of T4Δ*rrgB* and T4RΔ*rrgB* described above. Therefore, we examined RrgA topology in the unencapsulated strain T4RΔ*srtBCD*. This strain displayed RrgA foci with severely disorganized cellular localization, thereby confirming the essential role of the sortases for proper surface localization (Fig. S5).

Expression of *srtB* alone in T4Δ*srtBCD*+*lacE*::*srtB* is sufficient to restore surface detection of RrgB (Fig. 5G) as well as polymerization of RrgB and decoration with RrgA and RrgC (Fig. 2). Despite polymerization, RrgB (Fig. 5G) and RrgA (Fig. S5) was observed in many small, poorly organized foci in T4Δ*srtBCD*+*lacE*::*srtB*, verifying the phenotype of Δ*srtD*, as inactivation of *srtC* has no effect on pilin topology. In a similar fashion, the topology of RrgB and RrgA was examined in T4Δ*srtBCD*+*lacE*::*srtC*, expressing SrtC, but not SrtB or SrtD. Like T4Δ*srtB*, T4Δ*srtD* and T4Δ*srtBCD*+*lacE*::*srtB*, T4Δ*srtBCD*+*lacE*::*srtC* expresses irregular poorly organized RrgB and RrgA foci (Fig. 5H and Fig. S5).

In contrast, surface RrgB and RrgA was not detectable by IF in T4Δ*srtBCD*+*lacE*::*srtD* (Fig. 5I and Fig. S5), which lacks expression of *srtB* and *srtC*. This observation is corroborated by the WB data described above (Fig. 2A–C), wherein no high-molecular-weight RrgA, RrgB or RrgC polymers were detected in cell wall-associated protein fractions of T4Δ*srtBCD*+*lacE*::*srtD*. Thus, either SrtB or SrtC is necessary for polymerization and surface detection of RrgA and RrgB, but both SrtB and SrtD are necessary for wild-type surface localization.

To quantify the localization patterns found by IF, we examined single bacteria for localization and expression of pili. As shown in Table 1, 78% of pilus-positive wild-type T4 cells displayed regular, localized RrgB foci. Disruption of *srtB* or *srtD* reduced the fraction of well-organized foci to only 21% and 25%, respectively, confirming the essential roles of SrtB and SrtD for proper surface distribution of pili (Table 1). Complemention of the mutations *in trans* restored surface distribution to a value not significantly different from the wild-type T4 strain. Disruption of *srtC* did not show a statistically different proportion of regularly localized cells in comparison with the wild-type T4. Furthermore, cell populations of strains T4Δ*srtBCD*+*lacE*::*srtB* and T4Δ*srtBCD*+*lacE*::*srtC*, expressing only SrtB or SrtC, respectively, contained only 21% and 23% pilus-positive cells with symmetrical pairs of RrgB foci.

**Table 1:** Quantification of bacterial cells displaying localized or mislocalized pili and relative amount of bacteria expressing pili.

	Relative amount of pilus expressing bacteria with				
Strain	Localized pili	Dislocalized pili	*P*-value	Relative amount of bacteria expressing pili	*P*-value
T4Δ*srtB*	21.3 ± 5.2	78.7 ± 5.2	< 0001***	79.5 ± 9.6	< 0001***
T4Δ*srtC*	67.5 ± 4.7	32.5 ± 4.7	> 0.1 ns	86.7 ± 6.4	< 0001***
T4Δ*srtD*	24.5 ± 5.0	75.5 ± 5.0	< 0001***	87.4 ± 4.2	< 0001***
T4Δ*srtB*+*srtB*	77.7 ± 1.5	22.3 ± 1.5	> 0.1 ns	84.9 ± 2.6	< 0001***
T4Δ*srtC*+*srtC*	73.1 ± 6.7	26.9 ± 6.7	> 0.1 ns	87.3 ± 3.8	< 0001***
T4Δ*srtD*+*srtD*	78.6 ± 2.0	21.4 ± 2.0	> 0.1 ns	85.3 ± 4.1	< 0001***
T4Δ*srtBCD*	0	0	< 0001***	0	< 0001***
T4Δ*srtBCD*+*srtB*	21.2 ± 2.3	78.8 ± 2.3	< 0001***	76.6 ± 7.1	< 0.01**
T4Δ*srtBCD*+*srtC*	23.4 ± 2.8	76.6 ± 2.8	< 0001***	50.0 ± 2.9	> 0.1 ns
T4Δ*srtBCD*+*srtD*	0	0	< 0001***	0	< 0001***
T4Δ*srtB*+*srtB* (C>A)	26.9 ± 3.2	73.1 ± 3.2	< 0001***	75.7 ± 2.8	< 0.01**
T4	77.9 ± 4.5	22.1 ± 4.5	–	55.4 ± 7.9	–

Data are means and standard deviations of three independent experiments. For each experiment, samples were blinded and analysed concerning RrgB expression and localization. Pilus-positive bacteria were set to 100% to determine the relative amounts of bacteria with localized or mislocalized pili. Proper localization was defined as demonstration of symmetrical, paired foci (Fig. S7). Statistical significance of data was analysed by one-way anova and subsequent Bonferroni's multiple comparison test. The overall significance is *P* < 0.0001 for both analyses. Stated *P*-values were derived from comparison of the respective strain versus the T4 control.

Determination of the relative amounts of bacteria expressing pili within the cell population revealed that a lower fraction of wild-type T4 bacteria presented pili on the surface as compared with most mutant strains used in the study (Table 1). These data are in accordance with the WB data showing weak signals for pilin proteins using wild-type T4 (Fig. 2). The reason is unknown, but it is possible that the presence of the used antibiotic resistance cassette in mutant strains might affect pilus expression. However this difference had no effect on pilus topology, as the piliated fraction of T4 cells exhibited localized pilus expression to the same high degree as the complemented *srtB*, *srtC* and *srtD* mutants (Table 1).

**Fig. 6:**
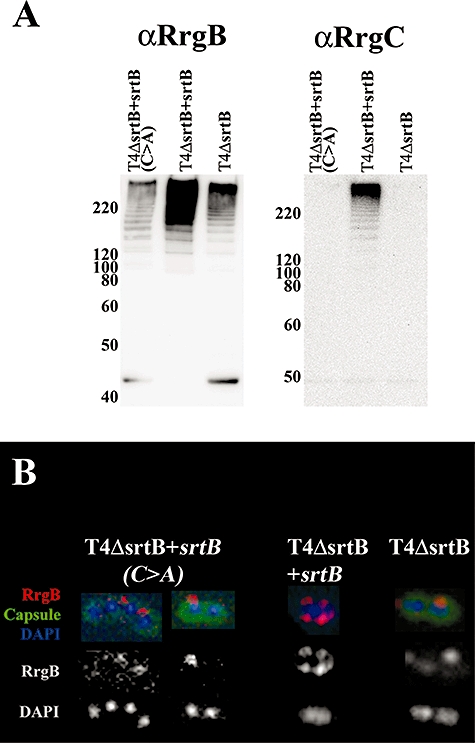
The active-site cysteine in SrtB is important for incorporation of RrgC into the pilus polymer in the presence of SrtC and SrtD. A. The role of the active-site cysteine of SrtB was investigated using a SrtB mutant where SrtB, with a substitution of the active-site cysteine for an alanine, was inserted in the *lacE* locus, in accordance with the other *trans*-complemented strains used in this study. B. The topology of RrgB was demonstrated to be affected by exchanging the active-site cysteine for an alanine whereby the regular pattern of foci found in the wild-type SrtB was exchanged to an irregular distribution.

Given that SrtD is essential for localization but dispensable for polymerization, it is possible that localization is mediated by protein–protein interactions between sortase enzymes, independent of enzymatic activities. It has been predicted that after cleavage of the LPXTG motif of pilin monomers the threonine binds to the cysteine residue of the transpeptidase whereafter the threonine is transferred to a lysine of the next pilin subunit (Ton-That and Schneewind, 2004). Hence, to elucidate if the enzymatic activity of SrtB, rather than the presence of the protein, is required for regular surface expression of pilus antigen, we changed the active-site cysteine into the residue alanine. As discussed above the enzymatic activity of SrtB can be assessed using WB analysis with RrgC antibodies. Pneumococcal cells expressing the mutated SrtB in the presence of intact SrtC and SrtD failed to incorporate RrgC into the RrgB polymer, thereby confirming the essential role of the cysteine residue for sortase activity (Fig. 6A). IF analysis with antibodies against RrgB revealed that the active-site cysteine mutant in SrtB presented completely disorganized foci in contrast to the regular pattern observed in cells expressing wild-type SrtB. Thus, only 27% of pilus-positive cells mutated in SrtB (cys>ala) displayed symmetrical RrgB foci, which is not significantly different from the *srtB* deletion mutant (21%, *P* > 0.05), but significantly different from the T4 strain expressing a functional SrtB *in trans* (78%, *P* < 0001) (Table 1). These data indicate that the proper surface distribution of pili is dependent on the enzymatic activity of SrtB (Fig. 6B).

## Discussion

This work describes the basic structural organization of subunits in the pneumococcal pilus, the cellular topology of pilins and the contributions of the pilus-associated sortases to both phenotypes.

Our studies suggest that pilus fibres are 2 nm in diameter, a value that matches those reported for the diphtherial (Ton-That and Schneewind, 2003) and streptococcal pili (Abbot *et al*., 2007). This pneumococcal pilus fibre is composed of RrgB monomers, based on several pieces of data. First, *rrgB* is necessary for production of high-molecular-weight species of all three pilins by WB of cell wall-associated proteins, and for production of extracellular fibres by AFM and EM. Second, anti-RrgB antibodies decorate pilus fibres along the entire length, in contrast to anti-RrgA and anti-RrgC antibodies (this work; Barocchi *et al*., 2006; LeMieux *et al*., 2006; Hilleringmann *et al*., 2008). Third, RrgB expression is necessary to detect RrgA by IF in the presence of a capsule, a further support for a critical role in structure. Finally, neither *rrgA* nor *rrgC* is necessary for pilus fibre formation, and both are localized to electron-dense peripheral patches and peripheral ‘knobs’.

Immunoelectron microscopy suggests that there are many ‘naked’ RrgB monomers, with a minority decorated with RrgA and RrgC in dense patches. However, complex arrangements with RrgA attached to RrgC or vice versa are suggested by WB of cell wall-associated proteins from *rrgB* mutants showing an RrgA–RrgC heterodimer, and similar ancillary subunit heterodimers have been reported in group B streptococcus pili upon deletion of the major pilin (Dramsi *et al*., 2006). The question of how and where ancillary subunits are conjugated to pilin polymers is relevant to the study of pathogenesis, as minor pilins have been repeatedly found to play important roles in adherence (Dramsi *et al*., 2006; Abbot *et al*., 2007; Krishnan *et al*., 2007; Maisey *et al*., 2007; Mandlik *et al*., 2007; Nelson *et al*., 2007) and virulence (Hava and Camilli, 2002).

It is clear that across all piliated Gram-positive bacteria, expression of pilus-associated sortases is required for pilus assembly, regardless of how many sortase genes are found in the pilus genetic locus (Yeung and Ragsdale, 1997; Yeung *et al*., 1998; Ton-That and Schneewind, 2003; Ton-That *et al*., 2004; Mora *et al*., 2005; Dramsi *et al*., 2006; Gaspar and Ton-That, 2006; Nallapareddy *et al*., 2006; Rosini *et al*., 2006; Swierczynski and Ton-That, 2006; Mishra *et al*., 2007). Polymerization in single-sortase pilus systems is thereby dependent on expression of the associated sortase gene (Yeung *et al*., 1998; Ton-That and Schneewind, 2003; Barnett *et al*., 2004; Mora *et al*., 2005; Nallapareddy *et al*., 2006; Mishra *et al*., 2007), while polymerization is a redundant process in two-sortase pilus systems (Dramsi *et al*., 2006; Gaspar and Ton-That, 2006; Rosini *et al*., 2006), with decoration of the polymer with ancillary subunits accomplished by different enzymatic tactics (Dramsi *et al*., 2006; Gaspar and Ton-That, 2006; Rosini *et al*., 2006). The biosynthetic logic exhibited by the sortase enzymes of the pneumococcal pilus continues the theme of redundancy, with RrgB polymerization and RrgA decoration catalysed by SrtB or SrtC, although SrtB may play a dominant role. However, SrtB alone is responsible for the incorporation of RrgC, making this enzyme necessary and sufficient to assemble a mature pilus.

What structural and biochemical features permit SrtB to recognize and process all three pilus subunits, but leave SrtC and SrtD substrate restricted? SrtB is not notably different from the other two pneumococcal pilus-associated sortases by primary sequence analysis. Indeed, SrtD is more distant from SrtB and SrtC than the latter two are from one another (Comfort and Clubb, 2004), an observation that may be related to its apparent dispensability in pilin polymerization. Regardless, it is likely that subtle structure-function biology underlies the distinctive capacity of SrtB to efficiently polymerize RrgB and incorporate RrgC into pili, as compared with SrtC. Such subtleties are suggested by attempts to re-engineer the sorting-signal specificity of staphylococcal sortase A (Bentley *et al*., 2007). We show here that the active-site cysteine in SrtB is responsible for incorporation of RrgC into the pilus polymer as well as important for RrgB topology (see below). However, this active-site cysteine of SrtB does not affect polymerization of RrgB in cells expressing SrtC.

Polymerization of pilins is but one dimension of the maturation of a wild-type pilus, and appropriate surface localization is another. We demonstrate symmetric, regular organization of wild-type pili by surface IF, a novel observation made in piliated Gram-positive bacteria. Expression of enzymatically active SrtB, i.e. with an intact cysteine residue, is necessary for wild-type topological distribution on the pneumococcal cell surface in addition to actually effecting the polymerization of pilin gene products. Furthermore, *srtD* mutants also lose the ordered topology of pilus antigen presentation on the cell surface, despite the apparent production of complete RrgA-, RrgB- and RrgC-positive polymers. The means by which SrtB and SrtD collaborate to effect pilin localization is, as yet, unknown, and the subject of ongoing investigations.

Pilin topology, as determined by IF, does not reflect the distribution of mature fibres, as both AFM and EM failed to detect localized foci of pili. The foci observed by IF do not represent pilin monomers, however, as RrgA foci are not detected in encapsulated T4Δ*rrgB*, despite abundant monomers by WB. Therefore, we propose that the pilin antigen foci detected by IF represent a multitude of intermediate-length multimers of pilins, long enough to extend into or past the polysaccharide capsule, but too short to be well visualized by AFM or EM approaches. The existence of such intermediate-length multimers is supported by pilin oligomers on WB. Polymers creating visible fibres of 1 μm or more are expected to contain many hundreds of covalently associated pilin subunits and may therefore be too large to enter into SDS-PAGE.

Given that pilin antigen foci are likely to represent short multimers, and that they depend on the sortases for both polymerization and normal localization, we hypothesize that these foci reflect sites for pilus assembly. We further hypothesize that these sites contain sortase enzymes, such as SrtB and/or SrtD, to facilitate polymerization. Protein secretion has been shown to occur at specialized sites in *Streptococcus pyogenes* (Rosch and Caparon, 2004). In separate studies, polarized protein secretion and sorting in *S. pyogenes* is controlled by unidentified factors in the secretion signals of divergently secreted proteins (Carlsson *et al*., 2006). Indeed, the activity of staphylococcal sortase A has been shown to be limited to a distinct topological site on the bacterial cell surface (DeDent *et al*., 2007), and SrtA and SecA, a component of the protein secretion apparatus, colocalize in iEM studies of *Streptococcus mutans* (Hu *et al*., 2007). In *E. faecalis*, SrtA and the pilus-associated SrtC colocalize with SecA and the ExPortal, indicating co-ordination of secretion, pilin polymerization and sorting machinery in Gram-positive bacteria (A.L. Gau and S.J. Hultgren, pers. comm.). Based on these observations, it is tempting to speculate that pneumococcal SrtD may interact with SrtB to control localization of the assembly machinery at the plane of cellular fission.

In conclusion, this work characterizes the basic structure and surface distribution of pneumococcal pili. The roles played by each gene in the *rlrA* islet pilus structure, assembly and surface topology of pili in *S. pneumoniae* are determined. We demonstrate specificity as well as redundancy for the two pilus-subunit polymerases SrtB and SrtC and the central role for polymerase SrtB as well as the non-polymerase SrtD sortase in the localized presentation of pneumococcal pili at the bacterial cell surface. We suggest that the presentation of pili adhesins as RrgA close together in foci on the bacterial cell surface allow for multiple localized adhesion receptor interactions promoting bacterial binding to host cells.

## Experimental procedures

### Cell wall preparations and immunoblotting

Cell wall-associated proteins were isolated from genetically defined strains of *S. pneumoniae* and analysed as previously described (Barocchi *et al*., 2006).

### AFM imaging of bacteria

Bacteria were grown as described in *Supporting information*, washed in PBS and fixed in 4% paraformaldehyde in PBS. Fixed bacteria were washed in PBS and destilled water and spotted onto freshly cleaved mica slides Grade V-4 (SPI Supplies, USA) mounted onto glass microscope slides and allowed to air-dry at room temperature in a dust-free environment. Bacteria were imaged in air with the BioScope SZ (Veeco Instruments, Woodbury, NJ, USA) operated in the contact mode using V-shaped silicon nitride nanoprobe cantilevers MLCT-HW (Veeco) with a spring constant of 0.05 N m^−1^ (Jonas *et al*., 2007). Images were captured using NanoScope v6.13 (Veeco) and prepared in Adobe Photoshop (Adobe, San Jose, CA, USA).

Data analysis was performed with the scanning probe software WSxM (Nanotec Electronica, Spain) (Horcas *et al*., 2007). Only high-quality images, in which the pilus tips looked well preserved, were chosen for the analysis. To measure the pilus diameter, height profiles crossing the pilus shaft were taken at 23 sites, from which mean and standard deviation were calculated. To analyse the occurrence of ‘tip knobs’, the total number of detectable tips and the number of tips with ‘knobs’ were counted for D39∇ (*n* = 5), D39∇Δ*rrgA* (*n* = 7) and D39∇Δ*rrgC* (*n* = 9), and the percentage of ‘tip knobs’ was calculated for each. The ‘internal knob’ frequency was calculated by dividing by the sum total length of all pili on an image file for D39∇ (*n* = 5), D39∇Δ*rrgA* (*n* = 6) and D39∇Δ*rrgC* (*n* = 8). To describe the distributions in both analyses, the median, the minimum and the maximum were determined for each strain.

### EM and iEM

Immunogold electron microscopy of pilus expression in TIGR4 was performed as previously described (Barocchi *et al*., 2006). Briefly, bacteria were grown as described in *Supporting information*, washed, and fixed in 4% paraformaldehyde in PBS for 20 min at room temperature in the dark. Fixed bacteria were then washed, and 4 μl drops of re-suspension were incubated on carbon-coated formvar nickel grids for 5 min, and blocked with PBS containing 2% BSA and 2% gelatin. Staining with 1:30 dilutions of anti-RrgB, anti-RrgA or anti-RrgC in 0.1% gelatin and 0.1% BSA in PBS was performed overnight. Samples were washed before secondary protein A conjugated to 10 nm gold particles was added at 1:1000 dilution. For double staining, a secondary goat anti-mouse IgG conjugated with 5 nm gold particles was used. Samples were then washed, fixed again and stained with 1% uranyl acetate, before analysis in a Philips CM10 transmission electron microscope. Digital images were taken by a Megaview III camera and prepared in Adobe Photoshop.

### IF imaging of bacteria

Mid-log phase (OD_620_ = 0.4) pneumococci were grown in C+Y, washed, struck on glass slides and allowed to air-dry. Slides were washed in PBS and fixed in 3% paraformaldehyde for 15 min, washed again, and incubated for 1 h at 37°C with a 1:1000 dilution of mouse anti-RrgA or mouse anti-RrgB, and 1:500 dilution of rabbit antiserotype 4 typing serum in PBS, with 1% BSA and 0.1% saponin. After washing with PBS, slides were incubated with Cy3-labelled anti-mouse and Cy2-labelled anti-rabbit secondaries. Slides were washed, and stained with DAPI for 10 min at room temperature before coverslipping.

All imaging was performed at the Karolinska Institutet core Visualisation Facility at MTC (KIVIF). Confocal visualization of bacteria was performed using the UltraVIEW ERS system (Perkin Elmer, USA), which includes a motorized 200M Axiovert fluorescence microscope (Zeiss GmbH, Göttingen, Germany), a CSU22 Nipkow spinning disc (Yokogawa, Tokyo, Japan), a Märzhauser motorized XY-table (Wetzlar-Steindorf, Germany), and an ORCA ER cold CCD camera with a detector array corresponding to 1344 × 1024 pixels (Hamamatsu City, Japan). Laser sources for five-line laser illumination (an Argon-ion laser at 488 and 514 nm, a Krypton laser at 561 nm, and two solid state lasers at 405 and 647 nm, from Melles Griot). Conventional microscopy was performed on Leica (Wetzlar, Germany) fluorescence microscopes equipped with Hamamatsu digital cameras operated by HiPic software (Hamamatsu), and images were prepared in Adobe Photoshop.

### Quantification of localization studied by IF

Immunofluorescence imaging of bacteria was performed as described above with antibodies raised against RrgB. Prepared images of three independent experiments were blinded, and subsequently around 150 bacteria per image and experiment were examined for pilus expression. Single cells were grouped into bacteria without any pili and into bacteria expressing pili. Pilus-positive bacteria were subgrouped into bacteria demonstrating localized pili and bacteria exposing dislocalized pili. Proper localization was defined as one symmetrical pair of foci presented per cell, whereas all other localization patterns including diffuse distribution, unpaired foci or multiple foci were regarded and counted as dislocalized. Schematic examples for localization and dislocalization are shown in Fig. S7. Significance of data was analysed by one-way anova and subsequent Bonferroni's multiple comparison test.
