# Supplementary material for: Sortase-mediated assembly and surface topology of adhesive pneumococcal pili
Source: Mol Microbiol. 2008 Sep 17;70(3):595–607. doi: 10.1111/j.1365-2958.2008.06396.x (PMC2680257; doi:10.1111/j.1365-2958.2008.06396.x)

SUPPORTING INFORMATION for:

Sortase mediated assembly and surface topology of adhesive pneumococcal pili

Stefan Fälker, Aaron L. Nelson, Eva Morfeldt, Kristina Jonas, Kjell Hultenby, Johannes Ries, Öjar Melefors, Staffan Normark and Birgitta Henriques-Normark

SUPPORTING MATERIALS AND METHODS

Bacterial strains, media, and growth conditions

Pneumococcal strains used, including isogenic mutant derivatives, are described in Supporting Table S1. The insertion-deletion mutagenesis used for most strains as well as the strategy to achieve complementation *in trans* are described elsewhere (Barocchi et al., 2006; Lau et al., 2002). The primers used for each strain are noted in Table S1, and all primers are described in Supporting Table S2. Resulting strains were checked by PCR, sequencing, and immunogenicity, thereby also demonstrating a lack of polar effects of deletion of one subunit gene on expression of others.

Unless otherwise noted, bacteria were streaked from frozen stocks onto blood plates with appropriate selection for overnight growth, inoculated briefly into pre-warmed DS medium (Dextrose-Serum medium, OXOID Manual, 1990), and then DS was inoculated into pre-warmed C+Y medium to achieve an $O.D._{620}$=0.05. Cultures were permitted to grow to mid-log ($O.D._{620}$=0.4) at 37° without agitation before collection for experimentation. Bacterial medium was produced by the Karolinska Microbiology laboratory.

SUPPORTING TABLES

Table S1. Characteristics of strains/mutants used in the study.

**Strain/mutant**	**Relevant characteristics**	**Primers employed in construction**	**Source/Reference**
T4	TIGR4 piliated invasive isolate	Not applicable	(Barocchi et al., 2006)
T4Δ*rrgA*	Erm^R, piliated strain lacking *rrgA*	Upstream: RrgA-1 and RrgA-2 Downstream: RrgA-3 and RrgA-4	(Nelson et al., 2007)
T4Δ*rrgB*	Erm^R, non-piliated strain lacking *rrgB*	Upstream: RrgB-1 and RrgB-2 Downstream: RrgB-3 and RrgB-4	This study
T4Δ*rrgC*	Erm^R, piliated strain lacking *rrgC*	Upstream: RrgC-1 and RrgC-2 Downstream: RrgC-3 and RrgC-4	This study
T4Δ*rrgABC*	Erm^R, non-piliated strain lacking *rrgA*, *rrgB,* and *rrgC*	Upstream: RrgA-1 and RrgA-2 Downstream: RrgC-3 and RrgC-4	This study
T4Δ*srtB*	Erm^R, piliated strain lacking *srtB*	Upstream: SrtB-1 and SrtB-2 Downstream: SrtB-3 and SrtB-4	This study

T4Δ*srtC*	Erm^R, piliated strain lacking *srtC*	Upstream: SrtC-1 and SrtC-2<br>Downstream: SrtC-3 and SrtC-4	This study
T4Δ*srtD*	Erm^R, piliated strain lacking *srtD*	Upstream: SrtD-1 and SrtD-2<br>Downstream: SrtD-3 and SrtD-4	This study
T4Δ*srtCD*	Kan^R, piliated strain lacking *srtC* and *srtD*	Upstream: SrtC-1 and SrtC-2<br>Downstream: SrtD-3 and SrtD-4	This study
T4Δ*srtBCD*	Erm^R, non-piliated strain lacking *srtB*, *srtC*, and *srtD*	Upstream: SrtB-1 and SrtB-2<br>Downstream: SrtD-3 and SrtD-4	This study
T4Δ(*rrgA-srtD*)	Erm^R, non-piliated strain lacking *rrgA*, *rrgB, rrgC, srtB, srtC,* and *srtD*	Upstream: RrgA-1 and RrgA-2<br>Downstream: SrtD-3 and SrtD-4	(Barocchi et al., 2006)
T4Δ*mgrA*	Erm^R, piliated strain lacking *mgrA*	see reference	(Barocchi et al., 2003)
T4Δ*srtB* + *lacE*::*srtB*	Spc^R, Erm^R, piliated strain containing *srtB in trans*	Upstream: NlacEF and SpcR1<br>Coding sequence: SrtB-aad9 and Sp0479-SrtB<br>Downstream: NlacER and Sp0479R	This study

T4Δ*srtB* + *lacE*::*srtB*(Cys→Ala)	Spc^R, Erm^R, piliated strain containing *srtB in trans*, Cys177 changed to Ala	Upstream: NlacEF and SrtBC177Arev Downstream: NlacER and SrtBC177Afor fragments from T4Δ*srtB* + *lacE*::*srtB* fused by overhang extension PCR	This study
T4Δ*srtC* + *lacE*::*srtC*	Spc^R, Erm^R, piliated strain containing *srtC in trans*	Upstream: NlacEF and SpcR1-EcoRI Coding sequence: SrtC5'-EcoRI and SrtC3'-HindIII Downstream: NlacER and Sp0479R-HindIII	This study
T4Δ*srtD* + *lacE*::*srtD*	Spc^R, Erm^R, non-piliated strain containing *srtD in trans*	Upstream: NlacEF and SpcR1-EcoRI Coding sequence: SrtD5'-EcoRI and SrtD3'-HindIII Downstream: NlacER and Sp0479R-HindIII	This study
T4Δ*srtBCD* + *lacE*::*srtB*	Spc^R, Erm^R, piliated triple-sortase mutant strain containing *srtB in trans*	NlacEF/NlacER fragment moved from T4Δ*srtB* + *lacE*::*srtB*	This study
T4Δ*srtBCD* + *lacE::srtC*	Spc^R, Erm^R, piliated triple-sortase mutant strain containing *srtC in trans*	NlacEF/NlacER fragment moved from T4Δ*srtC* + *lacE*::*srtC*	This study

T4Δ*srtBCD* + *lacE*::*srtD*	Spc^R, Erm^R, piliated triple-sortase mutant strain containing *srtD in trans*	NlacEF/NlacER fragment moved from T4Δ*srtD* + *lacE*::*srtD*	This study
T4R	Chloramphenicol^R, unencapsulated derivative of T4, piliated strain	Not applicable	(Fernebro et al, 2004)
T4RΔ*rrgB*	Erm^R, non-piliated strain lacking *rrgB*	RrgB-1/RrgB-4 fragment moved from T4Δ*rrgB*	This study
T4RΔ*srtBCD*	Erm^R, non-piliated strain lacking *srtB*, *srtC*, and *srtD*	SrtB-1/SrtD-4 fragment mover from T4Δ*srtBCD*	This study
D39	Non-piliated strain	Not applicable	(Iannelli et al., 1999)
D39∇(*rlrA*-*srtD*)	Spc^R, piliated strain containing the *rlrA* islet	RlrA-1/SrtD-4 fragment moved from T4	(Barocchi et al., 2006)
D39∇(*rlrA*-*srtD*) Δ*rrgA*	Spc^R, Erm^R, piliated strain lacking *rrgA*	RrgA-1/RrgA-4 fragment moved from T4Δ*rrgA*	This study
D39∇(*rlrA*-*srtD*) Δ*rrgB*	Spc^R, Erm^R, non-piliated strain lacking *rrgB*	RrgB-1/RrgB-4 fragment moved from T4Δ*rrgB*	This study
D39∇(*rlrA*-*srtD*) Δ*rrgC*	Spc^R, Erm^R, non-piliated strain lacking *rrgC*	RrgC-1/RrgC-4 fragment moved from T4Δ*rrgC*	This study

D39∇(*rlrA-srtD*) Δ*rrgABC*	Spc^R, Erm^R, non-piliated strain lacking *rrgABC*	RrgA-1/RrgC-4 fragment moved from T4Δ*rrgABC*	This study
BHN100 ($ST162^{19F}$)	colonizing isolate, piliated	Not applicable	(Sandgren et al., 2005)
BHN100 19FΔ*rrgA*	Erm^R, piliated strain lacking *rrgA*	RrgA-1/RrgA-4 fragment moved from T4Δ*rrgA*	This study
BHN100 19FΔ*srtB*	Erm^R, piliated strain lacking *srtB*	SrtB-1/SrtB-4 fragment moved from T4Δ*srtB*	This study
BHN100 19FΔ*srtD*	Erm^R, piliated strain lacking *srtD*	SrtD-1/SrtD-4 fragment moved from T4Δ*srtD*	This study

Table S2. Primers used in the study, with restriction enzymes sites.

Primer name	Sequence
RrgA-1	CAAGGTCCAAACCTACTGAAC
RrgA-2	GCGGGCCCCTGAGATATACAGCACAGTCC<br>*Apa*I site underlined
RrgA-3	CGGGATCCCCAGCGGGTTACGAGTTTAC<br>*Bam*HI site underlined
RrgA-4	CAACAAGGTGGAACACAGGTG
RrgB-1	GTGTAACAGGTCTGTACCTTG
RrgB-2	CGGGCCCGGTAACAGATGTTGTTGTCGTC<br>*Apa*I site underlined
RrgB-3	CGGGATCCGCATTACTAACTAGCCGTCAG<br>*Bam*HI site underlined
RrgB-4	GTCGTAACAGCATAGCCTGC
RrgC-1	GCTCAGACTGGTAAAGTTGTAC
RrgC-2	GCGGGCCCCCAACGTGTGATCTTCTTGCG<br>*Apa*I site underlined
RrgC-3	CGGATCCGGTCTAGAGTATGGGACATAC<br>*Bam*HI site underlined
RrgC-4	GCAATACCTCTTCAGCAGTAC
StrB-1	GTGGATGGTCGGACCAATAC
SrtB-2	GCGGGCCCCATACGTGCATACTCTGCTCG<br>*Apa*I site underlined
SrtB-3	CGGGATCCCAGTCATCTCTATCGCTACCTG<br>*Bam*HI site underlined
SrtB-4	GACCTTTCACCTGTCCAAGAG
SrtC-1	CGGAAAAGGCTTTGAAGGCG
SrtC-2	GCGGGCCCGGAAACCGTCTCATCAAACTC<br>*Apa*I site underlined
SrtC-3	CGGGATCCCAGTCATCGTCTGTTGGTACG<br>*Bam*HI site underlined
SrtC-4	CAGGATTCCCAGAAATGCCAG
SrtD-1	CGGAAAAGGCTTTGAAGGCG
SrtD-2	GCGGGCCCGACCTTTCACCTGTCCAAGAG<br>*Apa*I site underlined
SrtD-3	CGGGATCCCTGGCATTTCTGGGAATCCTG<br>*Bam*HI site underlined
SrtD-4	CGTTTCAAGTGCTATCACTGTTC
Erm-5'	TTTTTGGGCCCTTCGTGTTCGTGCTGACTTGC<br>*Apa*I site underlined

Erm-3'	TTTTTGGATCCGATGTTGCTGATTAAGACGAGC<br>*Bam*HI site underlined
NlacEF	GGGTATTGTGTGGATTAAAAAGG
NlacER	ACTGGTTTCTACAGGCTTGATTAG
SP0479R	GCTGTGTAGTAAGTTTTTCCA
SpcR1	CCCAGATCTCAATTTTTTTATAATTTTTTT
RrgB-aad9	GAGATTAAAAAAATTATAAAAAAATTGAGATCTGGGGATGTTTTCGAAAACTTGCAC
Sp0479-RrgB	TGGAAAAACTTACTACACAGCTTAAGCAAGTTGATCCTCATC
SpcR1-EcoRI	CGGAATTCCAATTTTTTTATAATTTTTTTAATCTG<br>*Eco*RI site underlined
Sp0479R-HindIII	CCCAAGCTTGCTGTGTAGTAAGTTTTTCCA<br>*Hind*III site underlined
SrtC5'-EcoRI	CGGAATTCGGAAAAGGCTTTGAAGGCG<br>*Eco*RI site underlined
SrtC3'-HindIII	CCCAAGCTTTTAGTCCTTGACATGACGCCC<br>*Hind*III site underlined
SrtD5'-EcoRI	CGGAATTCTATTACTAGGAGCGATGGCGG<br>*Eco*RI site underlined
SrtD3'-HindIII	CCCAAGCTTTTATTTCCCTCGTAGTAAACG<br>*Hind*III site underlined
SrtBC177A-for	GTGACCTTGCTGACTGCTACGCCATACATGATC
SrtBC177A-rev	GATCATGTATGGCGTAGCAGTCAGCAAGGTCAC

SUPPORTING FIGURE LEGENDS

Figure S1. Study of pilus expression in T4 and D39∇ populations by quantitative flow cytometry. Cultures of T4 and D39∇ were grown under identical conditions and prepared for measurement of RrgB expression by flow cytometry using a protocol modified from immunofluorescence studies. 10^5 cells from each culture were counted and RrgB expression, determined by Cy3-fluorescence, was plotted on a histogram. In addition, the T4 culture was also prepared in the absence of the anti-RrgB primary antibody, serving as a negative control ("negative"), confined entirely the first peak with low fluorescent intensity. The second peak, where the FL3 channel is scored 10^1 or greater (marked by a horizontal line above the peak), contains information about cells successfully stained for RrgB and detected. Note that more D39∇ cells were detected expressing RrgB than T4 cells. This finding suggests that a larger fraction of cells in a D39∇ culture express pili than the fraction of cells in a T4 culture. Genetic analysis of pilus polymer formation in D39∇ yielded identical results to those seen in T4, so D39∇ was used for ultrastructural analyses.

Figure S2. *rrgB* is required, and *rrgA* and *rrgC* are dispensable, for production of pilin polymers. Production of polymeric high-molecular weight extracellular pili in isogenic sets of mutants in T4 (A-C) and D39∇(TIGR4 *rlrA-srtD*) (D-F) were evaluated by preparation of cell wall-associated proteins and immunoblotting for pilus subunits. In all cases, cell wall proteins were separated by gradient SDS-PAGE, transferred to PVDF, and probed for RrgA (A, D), RrgB (B, E), and RrgC (C, F). Approximate molecular weights in kD are indicated on left, based on marker proteins. (A-C) Gel lanes were loaded with preparations from wild-type TIGR4 ("T4"), T4Δ*rrgA* ("Δ*rrgA*"), T4Δ*rrgB* ("Δ*rrgB*"), T4Δ*rrgC* ("Δ*rrgC*"), T4Δ(*rlrA-srtD*) ("Δ*rlrA-srtD*"). (D-F) Gel lanes were loaded with preparations from D39∇(TIGR4 *rlrA-*

srtD), ("D39+"), D39∇Δ*rrgA* ("Δ*rrgA"*), D39∇Δ*rrgB* ("Δ*rrgB"*), and D39∇Δ*rrgC* ("Δ*rrgC"*). These data demonstrate that extracellular pili are formed in the absence of *rrgA* and *rrgC* expression, but not in the absence of *rrgB*, in both T4 and D39∇(TIGR4 *rlrA-srtD*). In the absence of *rrgB*, the predicted 90 kD monomeric form of RrgA (indicated by asterisks 2) as well as a predicted 130 kD RrgA-RrgC heterodimer (asterisks 1) are incorporated into the cell wall in both serotypes. Moreover, incorporation of RrgA into polymeric structures does not require *rrgC*, and vice versa. Qualitatively identical results were obtained using the serotype 19F strain BHN100, of an independent clonal lineage from either T4 or D39∇(*rlrA-srtD*).

Figure S3. RrgA and RrgC are ancillary subunits independently incorporated into 'patches' along the length of pili. RrgB is the major pilin, as inactivation of the *rrgB* gene results in a lack of extracellular fibers, shown in D39∇(*rlrA-srtD*)Δ*rrgB* ("D39∇Δ*rrgB*") by EM (A, with 500 nm scale bar) and AFM (B, with 810 nm scale bar) analyses, and RrgB antigen is found widely distributed in D39∇(*rlrA-srtD*) ("D39∇") pilus fibers by iEM (C, 200 nm scale bar). (D-F) Pili are formed in strains with inactivated *rrgA* ("D39∇Δ*rrgA*"), by EM (D-E, 500 nm scale bars) and AFM (F, 1.0 µm scale bar). Inactivation of *rrgA* does not prevent incorporation of RrgC into pili (arrow in panel E, with 500 nm scale bar), although it does prevent detection of RrgA (D). (G-I) Likewise, mutation of *rrgC* does not prevent pilus formation by EM (G, 200 nm scale bar; H, 500 nm scale bar) or AFM (I, 1.7 µm scale bar); nor is incorporation of RrgA disrupted (G), although RrgC detection is abolished (H). Both RrgC (J-K, 500 and 100 nm scale bars, respectively) and RrgA (L-M, 500 and 100 nm scale bars, respectively) are found in 'patches' along pilus fibers. RrgC is also found at the tips of D39∇ pili (J-K).

Figure S4. Staining controls for immunofluorescent detection of RrgB on the bacterial cell surface. Demonstration of the specificity of immunofluorescent imaging for topologic studies. In these images, RrgB immunostaining was colored red, capsule immunostaining was colored green, and the nucleoid was stained with DAPI and colored blue. While wild-type T4 cells exhibit discrete, regular RrgB-positive banding (first column, "WT"), pilus-negative T4Δ*rrgA-srtD* cells exhibit no detectable signal when stained with antibodies against RrgB and a Cy3-conjugated secondary (second column, "Δ*rrgA-srtD*"). To emphasize the absence of fluorescent signal in the absence of intact genes, brightness in the panel showing the RrgB channel has been artificially enhanced in Photoshop. Wild-type T4 cells were also stained in the absence of anti-RrgB primary antibody and no Cy3-positive signal was detected (third column, "-αRrgB"). Again, to emphasize the absence of fluorescent signal in the absence of primary antibody, brightness in the panel showing the RrgB channel has been artificially enhanced in Photoshop.

Figure S5. Genetic determinants of RrgA topology. Conventional IF microscopy was used to analyze RrgA topology in wild-type T4, T4Δ*rrgC*, T4Δ*srtB*, T4Δ*srtD*, T4Δ*srtB*+*lacE*::*srtB*, T4Δ*srtD*+*lacE*::*srtD,* T4RΔ*srtBCD,* T4Δ*srtBCD*+*lacE*::*srtB,* T4Δ*srtBCD*+*lacE*::*srtC,* and T4Δ*srtBCD*+*lacE*::*srtD*, with images labelled as such. RrgA immunostaining was colored red, capsule immunostaining was colored green, and the nucleoid was stained with DAPI and colored blue. Like RrgB, RrgA is found in discrete, symmetrical, heterogeneous distribution on the surface of T4 cells, as shown in Fig 3. Note that mutation of *rrgC* does not grossly alter the distribution of surface RrgA, as observed for RrgB in T4. However, *srtB* mutation disrupts coordination of RrgA foci, much like the effect observed for RrgB in T4Δ*srtB*. Complementation of *srtB* in *trans*, strain T4Δ*srtB*+*lacE*::*srtB*, restores discrete symmetrical RrgA distribution, as shown for RrgB localization. Mutation of *srtD* results in the formation

of a large number of small, poorly organized RrgA foci diffusely distributed along cell chains, also similar to the effect on RrgB. Complementation *in trans* restores the proper RrgA topology. Disruption of all three sortases in the unencapsulated T4RΔ*srtBCD* results in the loss of proper surface localization of RrgA. RrgA distribution on the surface of strains expressing only single sortases is also in accordance with RrgB distribution as shown in Fig. 5. Also note that *srtB* and *srtD* mutations have similar effects on RrgB and RrgA topology in the serotype 19F strain BHN100 shown in Fig S6.

Figure S6. Pilus antigen topology in the serotype 19F strain BHN100. IF microscopy of piliated pneumococci from the serotype 19F strain BHN100 reveals symmetric, discrete, non-homogenous RrgB and RrgA topology, consistent with 'bands' or 'rings'. (A) BHN100 cells were stained with anti-RrgB (left) and anti-RrgA (right) antibodies as described, and visualized by confocal microscopy. RrgB immunostaining was colored red, capsule immunostaining was colored green, and the nucleoid was stained with DAPI and colored blue. Note that both RrgB and RrgA were found in highly discrete areas, and furthermore, that the two antigens likely overlap. A similar example of RrgB distribution in BHN100 is shown in Fig 3A, and examples of RrgB (Figs 3-5) and RrgA (Fig 3C, S5) topology in T4 are provided. (B) Genetic determinants of RrgB topology in the 19F strain BHN100 were studied by conventional microscopy. Wild-type 19F BHN100 exhibits discrete RrgB 'banding', similar to that observed by confocal microscopy (A). In contrast, genetic disruption of either *srtB* ("19FΔ*srtB*") or *srtD* ("19FΔ*srtD*") results in a loss of discrete, organized foci. Instead, many smaller randomly distributed RrgB foci are observed, similar to the phenotype of T4Δ*srtD* (Fig 5C). (C) Genetic determinants of RrgA topology in 19F BHN100 were studied by conventional microscopy. Wild-type 19F BHN100 exhibits discrete RrgA 'banding', as

shown by confocal microscopy in panel (A), similar to RrgA distribution in T4 (Fig 3C). Genetic disruption of either *srtB* ("19FΔ*srtB*") or *srtD* ("19FΔ*srtD*") results in a distribution, similar to the effects on RrgB in this strain (B), and in T4 (Fig S5).

Figure S7. Schematic examples for cells with localized and dislocalized pili. Single cells of wild type T4 and mutant strains were examined and grouped into bacteria presenting localized or dislocalized pili to quantify localization. Proper localization was defined as the display of one pair of symmetrical foci on either side of the single cell. Schematic examples of localization are provided (A). (B) Schematic examples of bacterial cells showing a different distribution of pili including unpaired foci, multiple foci, or disperse distribution. Corresponding cells were classified as dislocalized in surface display of pili.

SUPPORTING FIGURES

Supporting Figure 1.

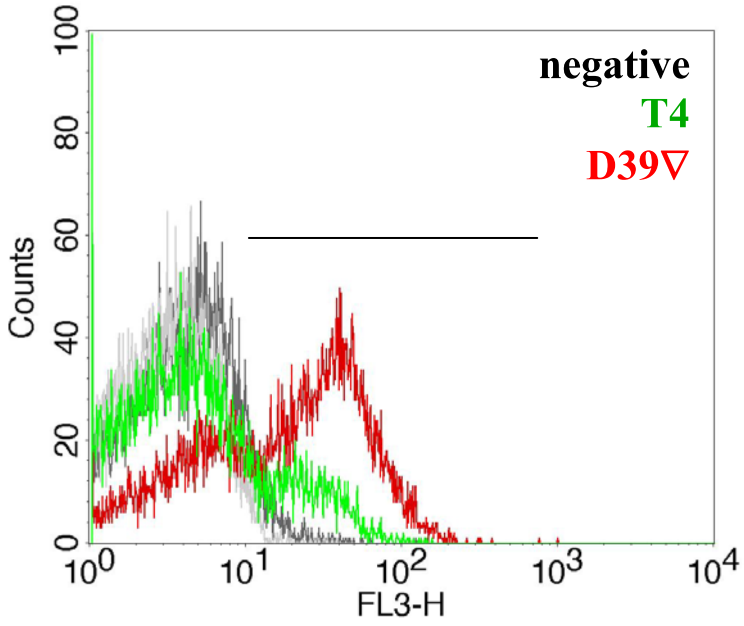

Supporting Figure 2.

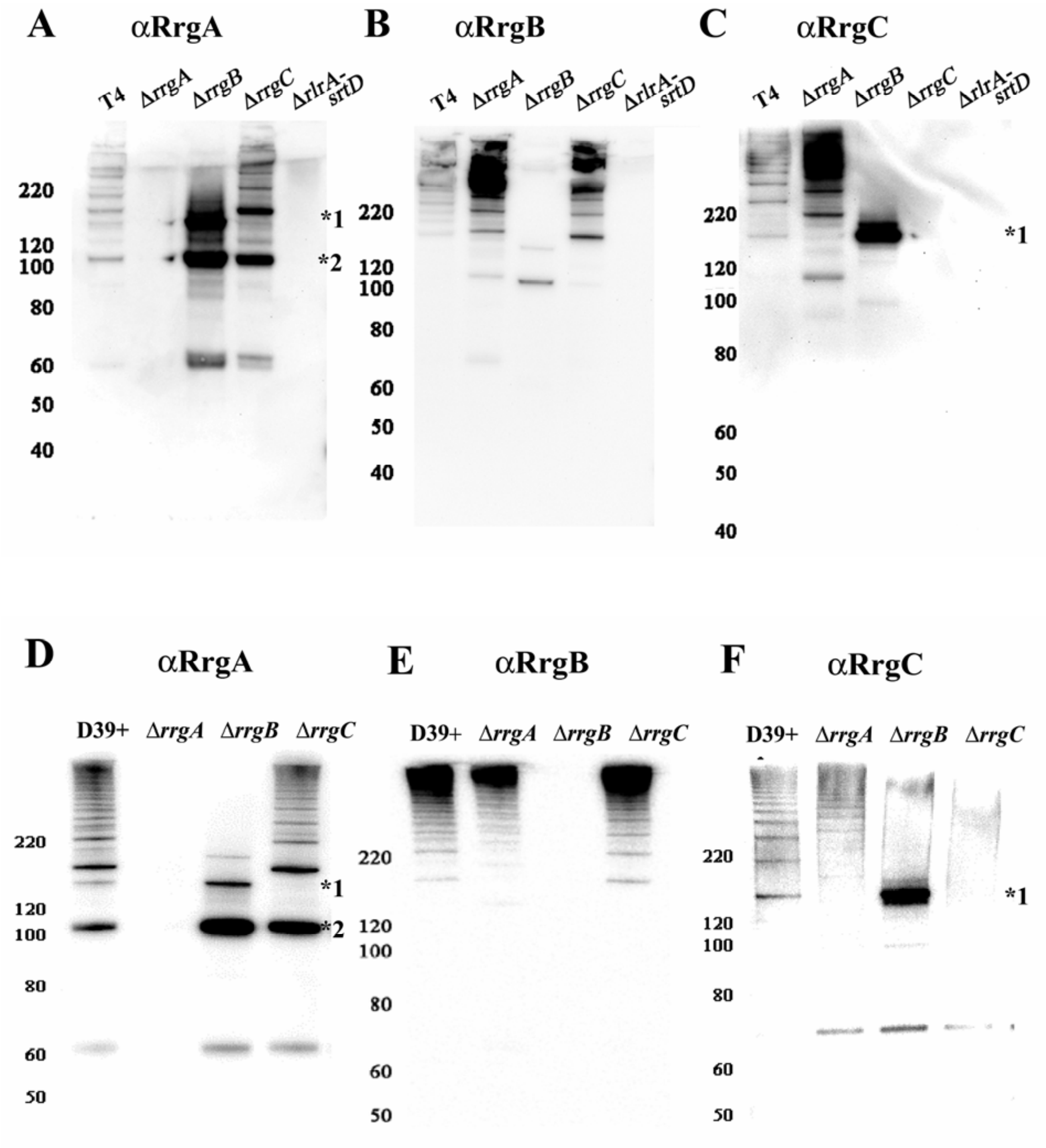

Supporting Figure 3.

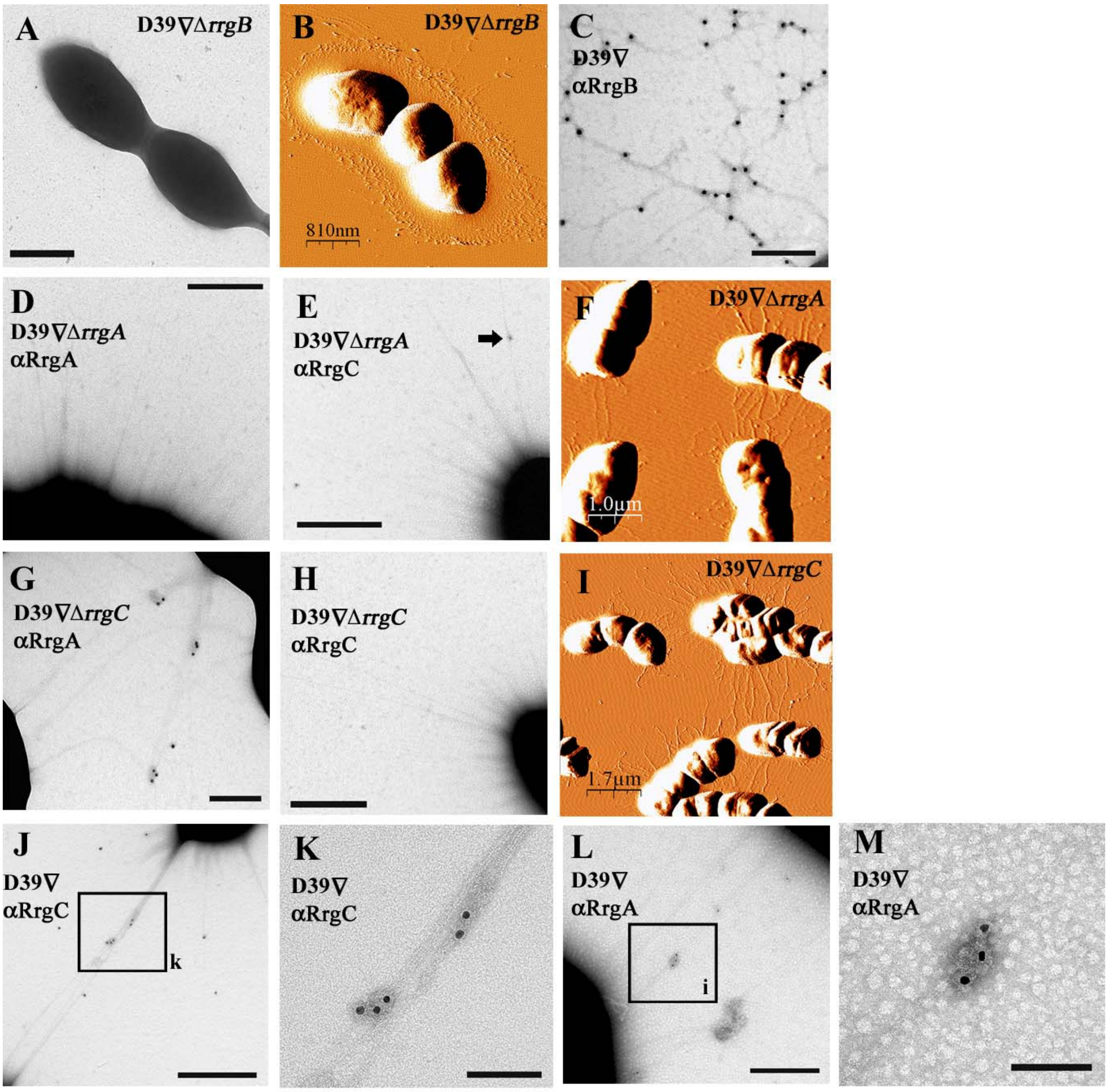

Supporting Figure 4.

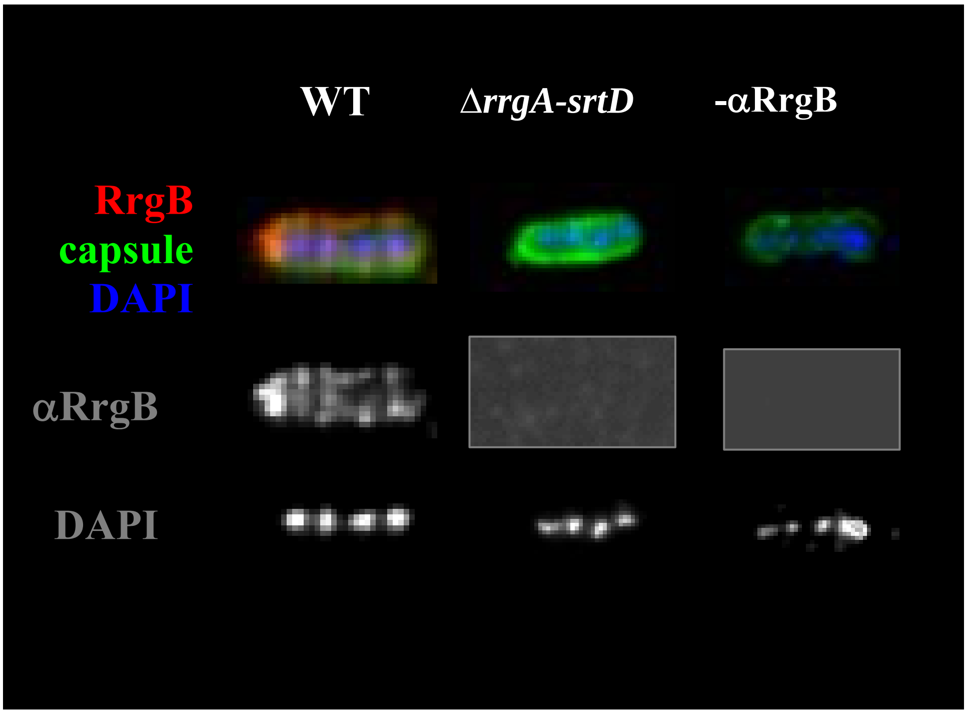

.

Supporting Figure 5.

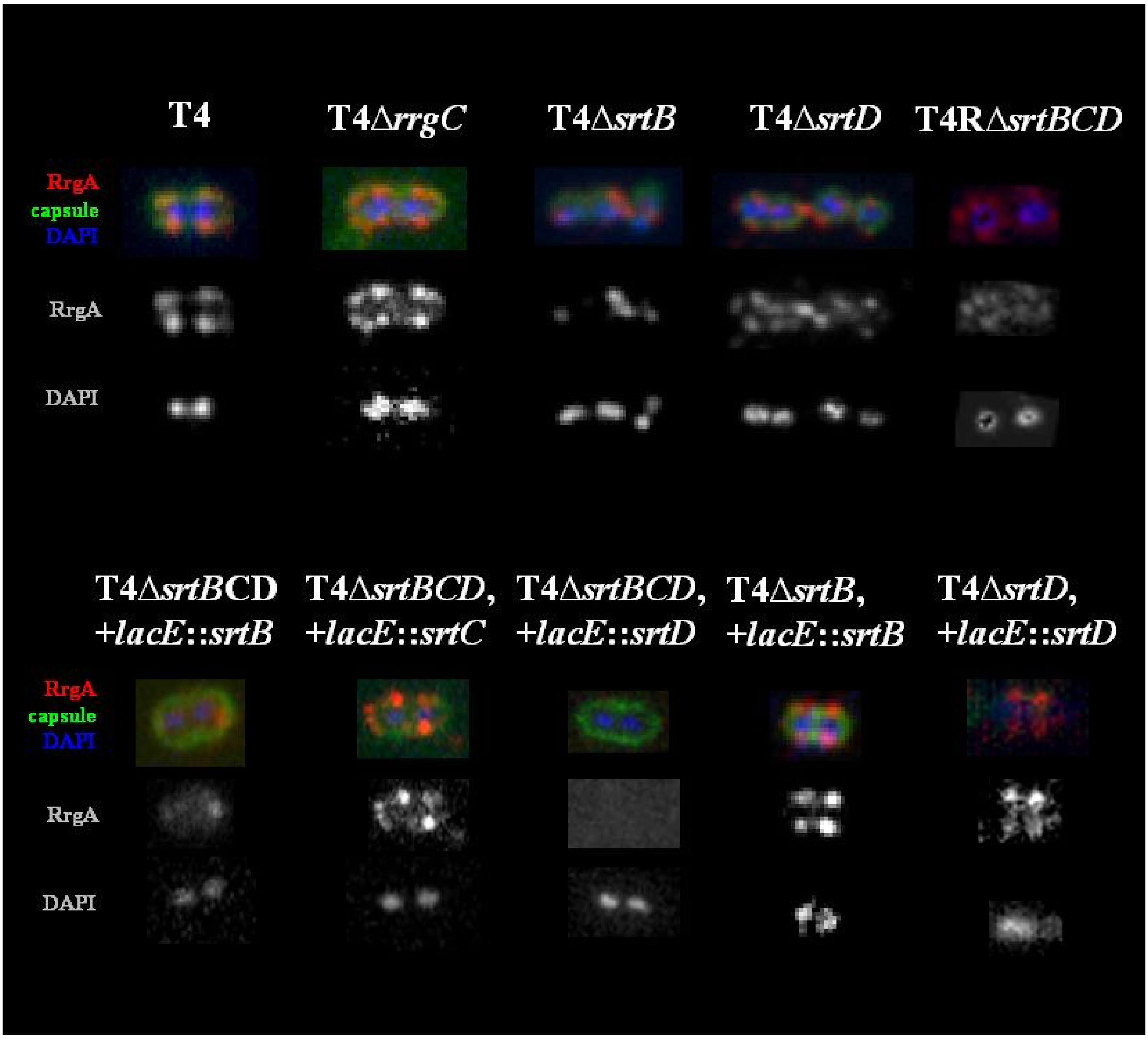

Supporting Figure 6.

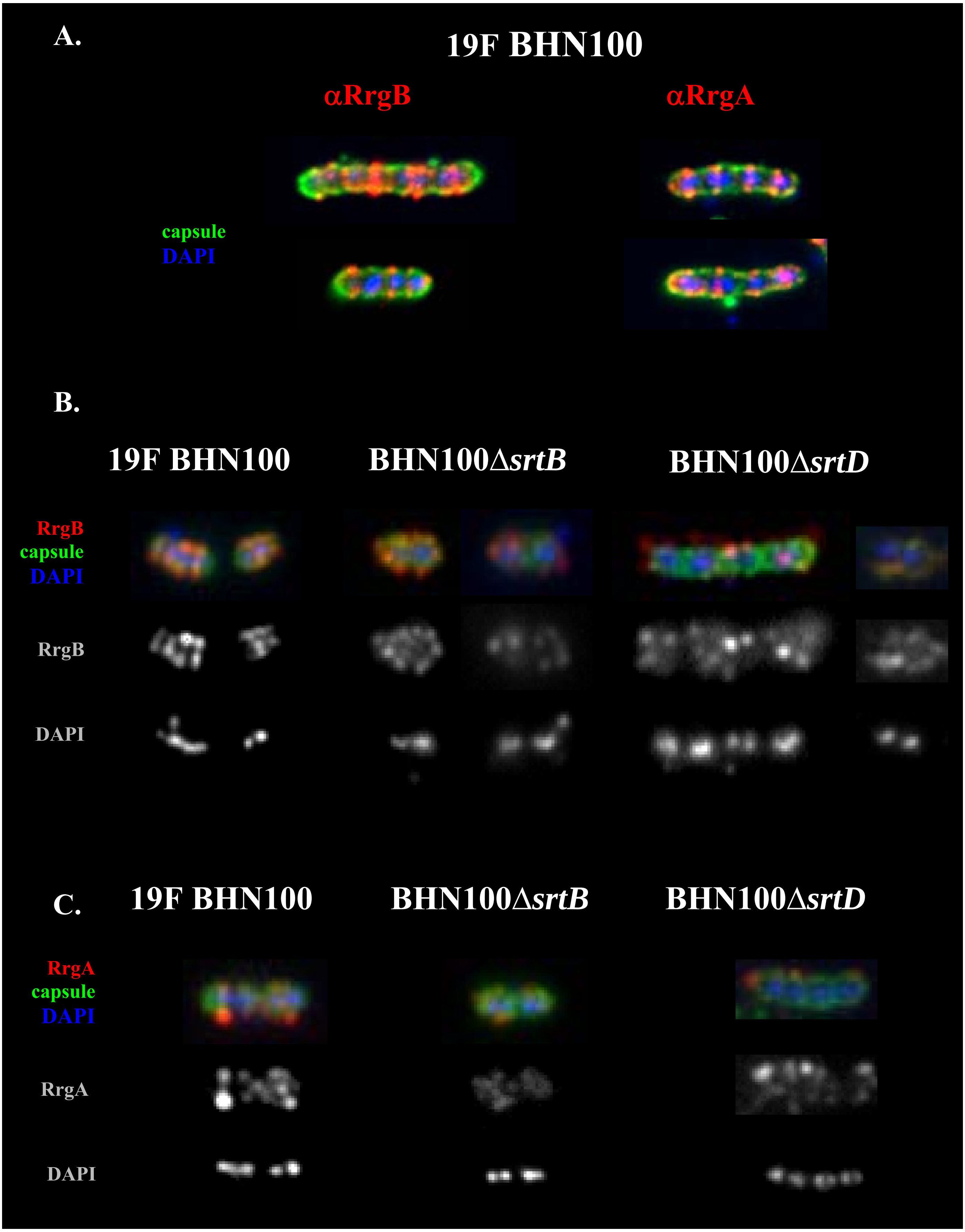

Supporting Figure 7.

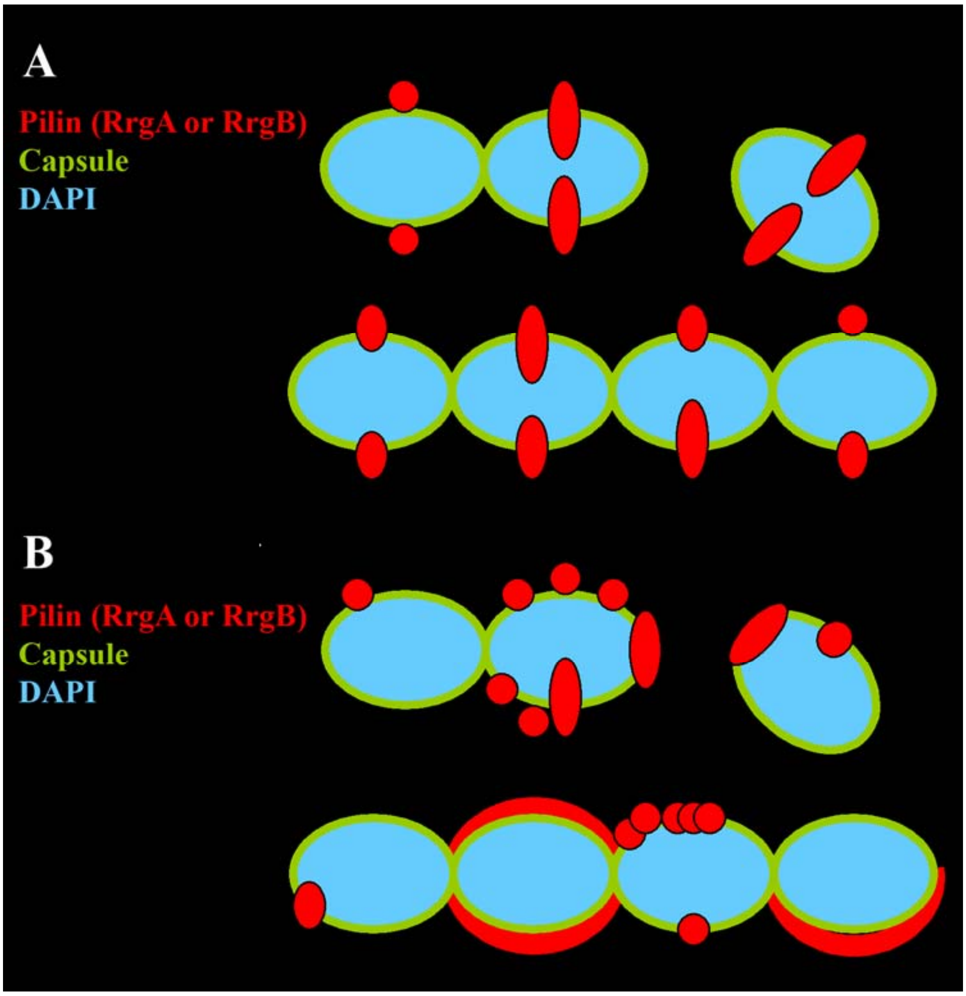

Supplement: Supplementary file 1 [file mmi0070-0595-SD1.pdf]
